# Supplementary material for: MetaRibo-Seq measures translation in microbiomes
Source: Nat Commun. 2020 Jun 29;11:3268. doi: 10.1038/s41467-020-17081-z (PMC7324362; doi:10.1038/s41467-020-17081-z)
Supplement: Supplementary file 10 — Supplementary Data 7 [file 41467_2020_17081_MOESM10_ESM.zip › File2/Confidence_High_Taxonomy/221177_out.krona.html]

Javascript must be enabled to view this page.

members
magnitude
magnitudeUnassigned
count
unassigned
taxon
rank

221177\_out

55

2
superkingdom
55

phylum
976
55

200643
class
55

55
171549
order

815
family
55

genus
816
55

species
47678
1

SRS019601\_contig\_number\_contig-100\_340.165702

821
species
2

SRS097920\_contig\_number\_14642SRS148784\_contig\_number\_2712

1263038
species

SRS013800\_contig\_number\_8657SRS065397\_contig\_number\_4971SRS893342\_contig\_number\_1449
3


SRS971275\_contig\_number\_14653
1
310298
species

1

SRS019787\_contig\_number\_3840
species
329854

162156
species

SRS065176\_contig\_number\_2630
1

46

SRS011239\_contig\_number\_3939SRS014235\_contig\_number\_26140SRS014459\_contig\_number\_28289SRS015065\_contig\_number\_3708SRS015579\_contig\_number\_14270SRS016018\_contig\_number\_8134SRS016095\_contig\_number\_3259SRS016203\_contig\_number\_3131SRS017191\_contig\_number\_12320SRS017701\_contig\_number\_4550SRS019030\_contig\_number\_17801SRS019267\_contig\_number\_1009SRS019445\_contig\_number\_24126SRS019685\_contig\_number\_23138SRS024435\_contig\_number\_24103SRS043701\_contig\_number\_10494SRS045713\_contig\_number\_9201SRS047014\_contig\_number\_contig-100\_3259.183459SRS049446\_contig\_number\_1983SRS053398\_contig\_number\_24848SRS053649\_contig\_number\_11223SRS054905\_contig\_number\_21286SRS058070\_contig\_number\_2708SRS063518\_contig\_number\_33244SRS075341\_contig\_number\_18788SRS075878\_contig\_number\_2432SRS075984\_contig\_number\_18507SRS076976\_contig\_number\_17920SRS097958\_contig\_number\_14600SRS098073\_contig\_number\_19SRS1041031\_contig\_number\_20934SRS1041092\_contig\_number\_3320SRS1041141\_contig\_number\_10298SRS104400\_contig\_number\_23706SRS104975\_contig\_number\_3231SRS1055034\_contig\_number\_8151SRS142923\_contig\_number\_15351SRS143342\_contig\_number\_6646SRS144537\_contig\_number\_contig-100\_1451.267947SRS145497\_contig\_number\_3962SRS146813\_contig\_number\_22948SRS148424\_contig\_number\_32441SRS148817\_contig\_number\_1086SRS149181\_contig\_number\_28833SRS149325\_contig\_number\_15890SRS893230\_contig\_number\_9683
species
246787
